# Class-aware feature attention-based semantic segmentation on hyperspectral images

**DOI:** 10.1371/journal.pone.0309997

**Published:** 2025-02-04

**Authors:** Prabu Sevugan, Venkatesan Rudhrakoti, Tai-hoon Kim, Megala Gunasekaran, Swarnalatha Purushotham, Ravikumar Chinthaginjala, Irfan Ahmad, Kumar A.

**Affiliations:** 1 Department of Banking Technology, Pondicherry University (A Central University), Puducherry, India; 2 School of Computing, SASTRA University, Thanjavur, India; 3 School of Electrical and Computer Engineering, Yeosu Campus, Chonnam National University, Yeosu-si, Jeollanam-do, Republic of Korea; 4 School of Computer Science and Engineering, Vellore Institute of Technology at Vellore, Vellore, India; 5 School of Electronics Engineering, Vellore Institute of Technology, Vellore, India; 6 Department of Clinical Laboratory Science, College of Applied Medical Sciences, King Khalid University, Abha, Saudi Arabia; 7 Data Science Research Laboratory, BlueCrest University, Monrovia, Liberia; National Textile University, PAKISTAN

## Abstract

This research explores an innovative approach to segment hyperspectral images. Aclass-aware feature-based attention approach is combined with an enhanced attention-based network, FAttNet is proposed to segment the hyperspectral images semantically. It is introduced to address challenges associated with inaccurate edge segmentation, diverse forms of target inconsistency, and suboptimal predictive efficacy encountered in traditional segmentation networks when applied to semantic segmentation tasks in hyperspectral images. First, the class-aware feature attention procedure is used to improve the extraction and processing of distinct types of semantic information. Subsequently, the spatial attention pyramid is employed in a parallel fashion to improve the correlation between spaces and extract context information from images at different scales. Finally, the segmentation results are refined using the encoder-decoder structure. It enhances precision in delineating distinct land cover patterns. The findings from the experiments demonstrate that FAttNet exhibits superior performance compared to established semantic segmentation networks commonly used. Specifically, on the GaoFen image dataset, FAttNet achieves a higher mean intersection over union (MIoU) of 77.03% and a segmentation accuracy of 87.26% surpassing the performance of the existing network.

## 1. Introduction

In the realm of remote sensing, the analysis of high-resolution images plays a pivotal role in extracting valuable insights for a myriad of applications, varying from urban planning to environmental monitoring. Semantic segmentation, a basic work in image analysis, holds the key to unlocking detailed information about the composition and characteristics of the observed scenes. However, the inherent challenges posed by high-resolution remote sensing images, such as intricate object details, scale variations, and complex contextual relationships, have underscored the need for advanced segmentation methodologies.

Traditional semantic segmentation methods often fall short in effectively addressing these challenges, leading to inaccuracies and suboptimal results. Recognizing this gap, our study focuses on refining the semantic segmentation process [1] through the incorporation of a class feature attention [2] mechanism. This innovative approach aims to enhance the discernment of unique class features within high-resolution images, offering a more nuanced and accurate representation of the observed scenes.

The resolution of remote sensing photos has been steadily increasing in recent years due to the ongoing advancements in remote sensing technology, which have led to the usage of an increasing number of high-resolution satellites. Automated analysis of complicated high-resolution hyperspectral images has become a critical concern. One of the most important technologies [3] for interpreting hyperspectral images is semantic segmentation [4], which has been applied extensively in a variety of industries such as environmental monitoring, change detection, urban planning, and land cover mapping. Understanding an image at the pixel level, or classifying each pixel, is known as semantic segmentation. This process divides the image into several relevant targets, each of which is given a particular label type. Extensive data on ground items can be obtained from high-resolution remote sensing photographs, which can also precisely depict the texture and spatial organization of the objects. These extensive contextual information is useful to perform Semantic segmentation [5]. However, the semantic segmentation of remote sensing images [6] has faced enormous hurdles due to the wide scale, rich characteristics, and complicated and diverse information.

Conventional satellite image segmentation techniques [7] primarily extract the image’s low-level attributes, such as color, grayscale, spatial texture, and geometric qualities, and use those features to split an image into many mutually disjoint sections. While there are notable differences between the low-level traits in different locations, they exhibit consistency or uniformity within the same region. For example, the Otsu threshold segmentation algorithm separates the image into distinct areas based on a predetermined threshold; Traditional satellite image segmentation techniques primarily extract the image’s low-level attributes, such as color, grayscale, spatial texture, and geometric qualities, and use those features to split an image into many mutually disjoint sections. While there are notable differences between the low-level traits in different locations, they exhibit consistency or uniformity within the same region.

Machine learning techniques are presented to perform semantic segmentation on hyperspectral images to the widespread adoption of deep learning algorithms [8]. Basaeed et al. (2016) [9] classified supervised pixels in spatial and spectral remote sensing pictures using the enhanced support vector machines (SVM) technique. The issue of the supervised classification’s lack of labeled pixels was resolved by this technique, which segmented several land cover categories using a modest amount of labeled pixels. The Markov arbitrary field technique using a tree-based arrangement was employed by Poggi et al. [10] to oversee the segmentation results on hyperspectral images. The algorithm’s overall accuracy in hyperspectral image segmentation was 86.5%. An enhanced conditional arbitrary fields technique was presented by Zhang et al. [11] to segment the synthetic aperture radar (SAR) images.

Deep learning has revolutionized the field of image processing and computer vision, leading to significant advancements in tasks such as classification, object detection, and semantic segmentation. Early models like AlexNet and VGGNet demonstrated the potential of deep convolutional neural networks (CNNs) in extracting hierarchical features from images. Subsequent models, such as ResNet, introduced residual connections, allowing for the training of much deeper networks without the problem of vanishing gradients. These advancements paved the way for more sophisticated architectures like U-Net and the DeepLab series, which incorporated encoder-decoder structures to enhance image segmentation tasks.

In recent years, attention mechanisms have emerged as a transformative addition to deep learning models, significantly improving their performance by enabling the network to focus on the most relevant parts of the input data. The attention mechanism [12], initially popularized by the Transformer model in natural language processing, has been successfully adapted to vision tasks. Models like the Vision Transformer (ViT) and attention-augmented convolutional networks have demonstrated the power of attention in capturing long-range dependencies and contextual information, which are often missed by traditional convolutional operations.

The integration of attention mechanisms into deep learning models offers several benefits [13, 14]. It allows the network to dynamically prioritize the most important features and regions of an image, leading to more accurate and context-aware predictions. In the context of semantic segmentation, attention mechanisms help in precisely delineating object boundaries and understanding complex spatial relationships, which are crucial for high-resolution remote sensing images. By incorporating class-aware feature attention, our approach further enhances the model’s ability to differentiate between various classes and capture fine-grained details, ultimately improving the segmentation accuracy and robustness in challenging environments.

Traditional methods often struggle to capture intricate details and differentiate between classes effectively. This issue hampers the accuracy and reliability of the segmentation process, impacting the quality of information extracted from the images. To overcome these challenges, the study proposes the utilization of a class feature attention mechanism. The problem at hand is to enhance semantic segmentation by incorporating a more sophisticated approach that focuses on the unique characteristics of each class within high-resolution remote sensing images. This involves addressing issues such as occlusion, scale variation, and complex contextual relationships [15] among different objects in the scene.

The significance of this problem lies in the potential applications of high-resolution remote sensing, where precise and detailed segmentation is crucial for tasks such as land cover mapping, urban planning, environmental monitoring, and disaster management. By devising a solution that integrates a class feature attention mechanism, this study aims to improve the accuracy and efficiency of semantic segmentation in this domain.

The motivation for this research stems from the critical importance of precise semantic segmentation in applications like land cover mapping, infrastructure development, and disaster response. The ability to distinguish between objects and classes with a higher level of detail not only refines our understanding of the observed environment but also empowers decision-making processes in various domains.

The major contributions of this research are as follows:

- A novel class-aware feature attention mechanism (FAttNet) is proposed to enhance the semantic segmentation of hyperspectral images by explicitly modeling the relationships between classes and feature channels. This mechanism allows the model to gather contextually relevant data, improving segmentation accuracy and robustness.
- By leveraging an encoder-decoder structure, our method effectively addresses the challenges associated with segmenting margin regions in high-resolution images. This architecture enhances the model’s ability to capture detailed spatial information and improve segmentation outcomes.
- The proposed approach significantly improves the mean Intersection over Union (mIoU) and overall segmentation accuracy on high-resolution remote sensing images.

The structure of the research article is as follows: Section 1 discusses the introduction of attention mechanism for hyperspectral image segmentation. Section 2 presents the related works, Section 3 represents the materials and methods used, Section 4 discusses the experimental result analysis made on benchmark dataset and the conclusion is discussed in Section 5.

## 2. Related works

Classification algorithms can be used to classify images using features such as color, texture, shape, and size. The accuracy of the classification results depends on the quality and availability of the training data. Regular testing and updating of the training data is necessary to ensure the accuracy of the results. This classification algorithm also assigns labels to the segmented region of the image. Fine-grained classification [16] can be used to classify objects within an image, such as detecting faces or animals. This approach requires an additional step of segmentation, where the image is divided into regions with similar features. Finally, object-level classification can be used to group similar objects into categories, such as recognizing different types of cars in a parking lot. Multi-class image-level classification can be used to better classify the different types of land cover in an image, allowing more accurate mapping and decision-making. Additionally, multi-class image-level classification can also be used to detect changes in land use over time. The feature extraction phase is combined with machine learning approaches to recognize and perfectly segment various land covers. In semantic segmentation, the pixel-wise classification approach is distinct from the whole image classification approach. In contrast to image-level classification [17], semantic segmentation assigns a label to each pixel in an image, resulting in an accurate and highly detailed representation of land cover types. This more accurate representation allows for better analysis of land cover dynamics and enables more precise land management decisions. Semantic segmentation is also required for applications such as autonomous vehicles, which require the ability to recognize and classify objects in 3D space.

Benchmark datasets are essential for the advancement of machine learning and artificial intelligence. Such datasets are carefully chosen to represent real-world problems, and are used to evaluate the performance of algorithms, models, and techniques. The good benchmark datasets are the UC Merced datasets, EuroSAT [18] and GaoFen Image datasets. These datasets are commonly used to assess the performance of object detection, image classification, and image segmentation algorithms [19, 20]. They also provide a comprehensive benchmark for researchers to compare the performance of different algorithms.

### 2.1 Encoder and decoder

Typically, an encoder lowers the resolution of feature maps to acquire semantic knowledge and broaden the receptive fields. The decoder subsequently reinstates the feature maps to their initial resolution, generating the final segmentation outcomes. Many studies, notably DeconvNet [21], update the upsample methods based on FCN [5] to produce improved results. Deconvolution is used in the decoding process by the DeconvNet [22]. Recently, Deep Convolutional neural network [23] has incorporateddual contextual components into distinct decoder network components, simulating feature interdependence in channels with spatial dimensions, individually.

Furthermore, multiple-stage feature [24] integration in encoder-decoder architecture is an active research topic. The UNet [21] progressively combines high and low level resolution information with low and high-level resolution characteristics, allowing spatial information across shallow layers to be used. It aids the decoder in producing better results. Many works [25, 26] use the U-Net [27] architecture. MoE-SPNet [28] provides a dynamic multilayer feature consolidation approach that uses skip-connections to add context. RefineNet [25] gathers all available information throughout the downsampling process, allowing enabling high-resolution forecasts employing long-term residual connections.

Dense Neural Network [29] extends CNN to deal with contextual segmentation despite any additional post-processing modules or pretraining. The approach proposed in Stacked Lossless Deconvolutions [26] incorporates additional oversights at each step to guide low-level features for increased semantic cues and high-level features for enhanced spatial details. Additionally, certain initiatives involve the stacking of multiple ResNet-152 [20] architectures to augment the network’s learning capabilities. Wang et al., [30] utilizes an encoder-decoder stack to aggregate diverse information. In the case of DCDN [6], multiple less-dimensional deconvolutional networks [31] are layered to deepen the construction and offer precise information for localization recovery. To improve feature fusion, ontologyreasons [32]are added with several unit connections to several deconvolutional networks. For effectively integrating the multiple scales background information, the researcher [33] presents a two phases encoder-decoder network connected with an attention module.

While the encoder-decoder architecture consistently delivers exceptional performance, addressing the disparity between deep and shallow features remains a challenge when relying solely on a fundamental encoder-decoder structure.

DeepLabv1-v3 serves as the foundation for DeepLabv3+ [34]. By including a decoder architecture to improve the object edge’s segmentation results, DeepLabv3+ expands on the features of DeepLabv3. Deeplabv1 undertakes complex classification assignments through a Deep Convolution Neural Network employing dilation rate in convolution. While this network generates a coarse prediction map, it incorporates a Conditional Random Field to refine segmentation outcomes. In contrast, Deeplabv1 faces challenges in multi-scale target segmentation. Deeplabv2 addresses this limitation by utilizing the Spatial Pyramid Pooling module, enhancing the capture of contextual information in images through parallel atrous convolution sampling with varying dilation rates on a specific feature layer.

### 2.2Attention mechanism

The attention mechanism is basically a matrix multiplication operation that can identify each pixel’s reliance relation in an image and boost the weights of the highly dependent pixels to reduce noise interference. This paper presents the feature attention module to improve the network’s understanding of each class’s information and more precisely describe the dependencies between classes. The class feature attention explicitly defines the connections between all classes within the image and all the channels. This capability enables the model to collect contextually relevant data from a remote perspective, considering the specifics of class information. It is possible to improve the contextual information interdependence among all classes.

Simultaneously, Deeplabv2 changes its initial layer from VGG16Net to ResNet-152 and incorporates a conditional arbitrary field to enhance the segmentation in the future. But if a 3 x 3 atrous convolution in spatial attention pyramid pooling adapts a higher dilation rate, it may degrade into a 1×1 convolution, resulting in the loss of long-distant information from the image. Deeplabv3 enhances the spatial attention pyramid module further, which includes a global average pooling layer in parallel, three 3 x 3 convolutions with dilation rates of 5,6, 11, and 12 and a 1x1 convolution. This modification aims to maintain a bigger receptive field while simultaneously addressing segmentation inaccuracies.

## 3. Materials and methods

**Fig 1:**
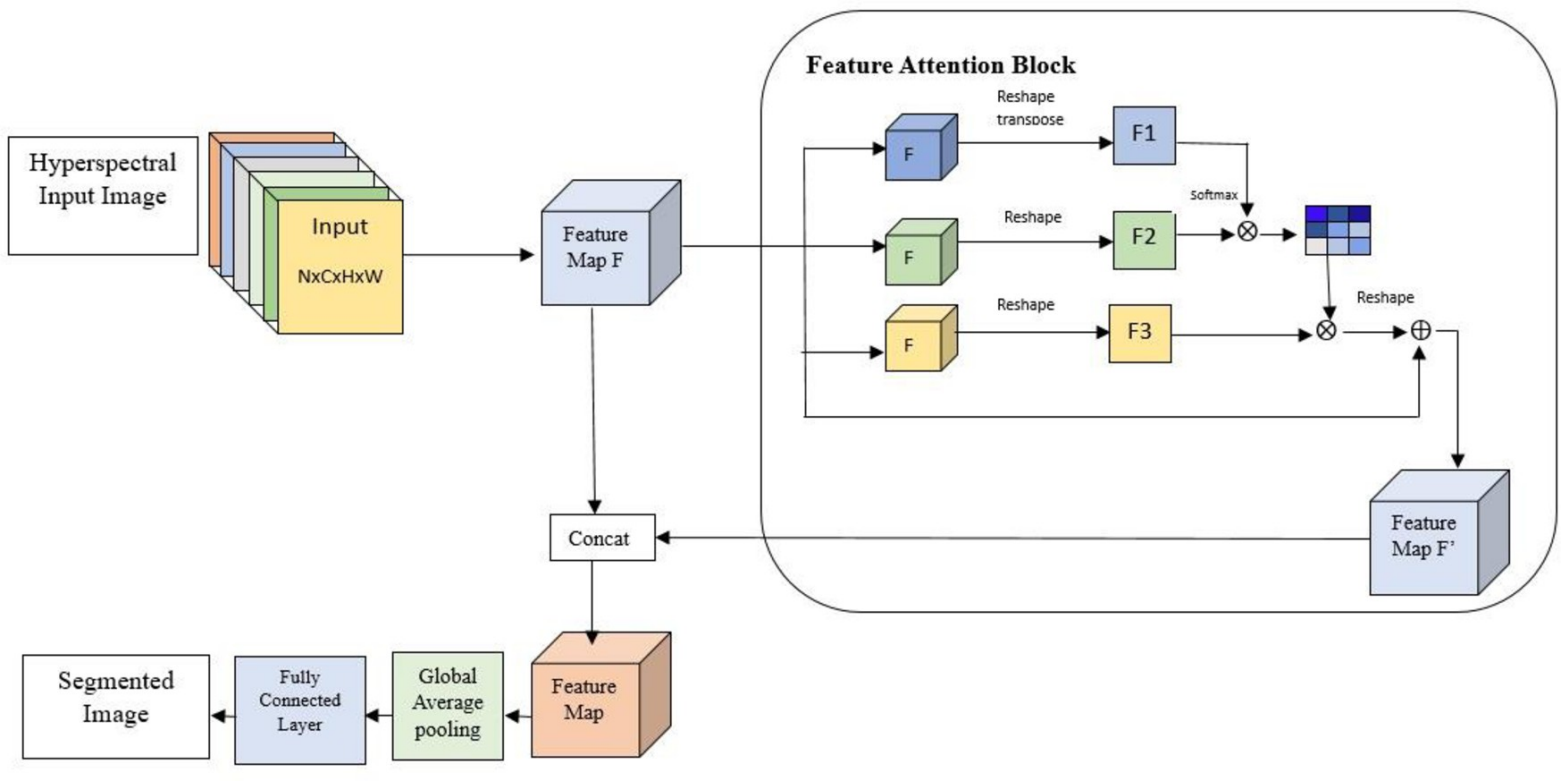
Feature-based attention network model.

The proposed research offers the Class aware FATTNet, an upgraded Deeplabv3+ network that employs the feature attention. The FAttNet module is responsible for extracting and processing context information among classes, while the spatial attention pyramid module focuses on extracting and processing spatial context information. This spatial context information is particularly effective in delineating various ground-based features in high-resolution remote sensing images. To optimize the segmentation of margin regions, FAttNet employs an encoder-decoder structure. A visual representation of the proposed attention mechanism can be observed in **Fig 1**.

The input image is passed to the encoder. The encoder Consists of 5 encoder blocks. Each encoder block consists of two convolutional 2D layers and a max-pooling layer to downsample the feature maps. The number of filters for each block increases sequentially: 64, 128, 256, 512, 512. Class Feature Attention Mechanism uses global average pooling and dense layers to generate attention maps. Global Average Pooling extracts global context information from the feature maps. Fully Connected Layers capture class-specific features using two dense layers (both with 512 units and ReLU activation). The final dense layer (with sigmoid activation) produces attention maps, which are reshaped and multiplied with the encoder’s output to emphasize class-specific features. The output is passed to the Spatial Attention Pyramid Module which performs atrous convolution, global average pooling and concatenation. Three parallel Conv2D layers with dilation rates of 6, 12, and 18 respectively, are done in atrous convolution function to capture multi-scale context information. Atrous convolution layers with different dilation rates are applied to the attention maps.Another global average pooling layer followed by dense layers is used to refine spatial features. Concatenation function Combines the output of atrous convolutions and global pooling to form the final feature map for the decoder. It is then passed to the decoder. The decoder consists of five decoder blocks. Each block consists of an UpSampling2D layer to upsample the feature maps and two Conv2D layers (with ReLU activation and ‘same’ padding) to refine the upsampled features. The output layer performs a final 1x1 convolutional layer with a softmax activation to produce the segmentation mask with 6 classes.

### 3.1 Class aware feature attention mechanism

On applying 1x1 convolution to the feature map of input image (A) by reducing the channel, then the new feature map $A^{\prime }\in R^{C^{\prime }\times H\times W}$ is obtained. Then the cross layered class-based feature map (E) is obtained by applying 1x1 convolution where *EϵR*^*N*×*H*×*W*^. The new feature map A’ is reshaped to the dimension of $R^{C^{\prime }\times HW}$. Softmax function (S) is employed to the resultant feature map. Dot matrix multiplication is performed on the transpose of A’ and E. The class-aware feature intricate matrix is computed as

$$CF_{n,k}=\sum_{i=1}^{HW}A_{n,i}^{\prime }\frac{e^{E_{j,k}}}{\sum_{j=1}^{N}e^{E_{j,k}}}$$ (1)

Where $A_{n,i}^{\prime }$ represents the nth channel of the feature map A’.*E*_*j*,*k*_ represents the ith pixel with k channel of the feature map E. The steps involved in Feature based attention to perform segmentation is illustrated in Algorithm 1.

The output feature maps are passed to the 1x1 convolution function followed by GPU synchronized Batch normalization and RELU activation function. The final representation of the class aware features is the weighted addition of all channels attention-based feature map.

Different sampling rates such as (3,6,12) are introduced to the spatial pyramid attention. Which helps to accurately locate the pixel. These different dilation rates (3,6,12) introduced to the kernel helps to preserve the long range interdependencies among the pixel. It also involves 3x3 convolutions, global average pooling in a parallel fashion.

### Algorithm 1: Feature Attention based Segmentation

Step 1: Initialize the FAttNet model with N number of classes and a pre-trained ResNet152.
Step 2: Forward input image to the network.
Step 3: Extract hierarchical features using the backbone network.
Step 3.1: Compute Attention weights by scaled dot-product attention
attention_weights = F.softmax(class_features,dim = 1)
Step 4: Compute class-specific features with spatial attention mechanism in the encoder layer.
Step 5: Perform convolutions, matrix multiplication and apply ReLU activation on the feature map.
Step 5: Fuse hierarchical and class-specific features.
Step 6: Generate the segmentation map.

**Fig 2:**
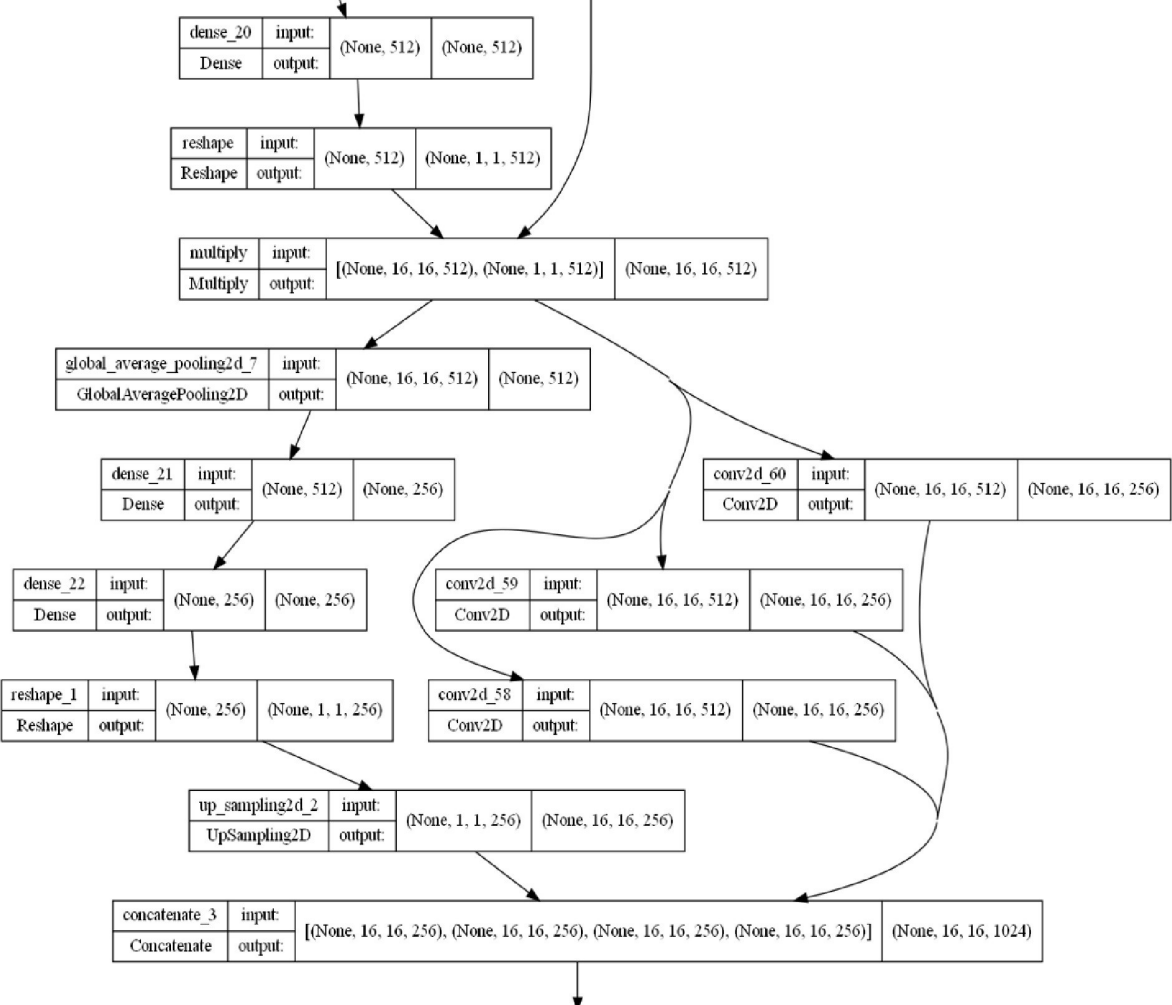
Spatial attention module of FAttNet model summary.

The FAttNet model is designed as a fully convolutional network (FCN) optimized for high-resolution remote sensing image segmentation. It incorporates a class-aware feature attention mechanism and an enhanced encoder-decoder architecture to achieve superior segmentation accuracy. The Spatial attention module which exists between the encoder block and decoder of the FAttNet model is shown in **Fig 2**.

## 4. Experimental analysis and results

Here, we test the proposed FAttNet network through a series of in-depth experiments on the GID and EUROSAT datasets, two rival datasets. A Linux system environment was used to conduct this experiment. Tensorflow 2.5 was the operating framework, and GPU acceleration was employed. The graphics card was an NVIDIA GeForce GTX 1650, and the server, an Intel(R) Core(TM) i7-processor CPU @ 3.00 GHz. Python 3.8.8 was the programming language used.

### 4.1 Dataset

The Land Use Classification dataset, known as EuroSATis made up of 27000 geo-referenced and annotated samples from 10 classes in 13 spectral bands from Sentinel-2 satellite pictures. Available in versions with 13 bands and RGB.

In this work, we employ Sentinel-2 satellite pictures to address the problem of classifying land use and land cover. The Sentinel-2 satellite photos are publicly available on Kaggle datasets named eurosat-dataset (https://www.kaggle.com/datasets/apollo2506/eurosat-dataset) as part of the Copernicus Earth observation program. This dataset comprised of 27,000 annotated and geo-referenced images across 10 classes, spanning 13 spectral bands and derived from Sentinel-2 satellite photographs. This dataset along with its spectral bands is passed to the proposed model with advanced deep Convolutional Neural Networks (CNNs) with an attention mechanism. We were able to obtain an overall classification accuracy of 98.57%. Many Earth observation applications are made possible by the resulting classification system. We show how land use and land cover changes can be detected using this classification method.

Gaofen-2 (GF-2) satellite is a comprehensive land-cover dataset comprised of satellite images. The recently established Gaofen Hyperspectral Image Dataset [35] surpasses existing land-cover datasets in terms of extensive coverage, widespread distribution, and high spatial resolution. This dataset comprises two main components: the fine land-cover classification set and the large-scale classification set. The fine classification set comprises 30,000 multi-scale image patches, each associated with 10 pixel-level annotated GF-2 images. Meanwhile, the large-scale classification set consists of 150 pixel-level annotated GF-2 images. For training and validation purposes, photos with 5 categories and data with 14 categories have been collected and re-labeled, respectively.

### 4.2 Implementation

Our backbone network is a Residual Net based on Fully Convolution network (FCN) with the dilation rates involved in convolution operation. For training both the datasets, the backbone’s output stride is set at 16. Bilinear interpolation is applied on each pixel to get the final segmentation by upsampling the feature maps to original size of the input image. Furthermore, PyTorch is used for all of our investigations.

Here the batch normalization function is replaced with GPU synchronized batch normalization for better variance approximation. Adam optimizer is used for optimizing the network along with a learning rate of 0.0005. 70K images are used for training and 30% of total images if used for validation. The summary of the model’s training parameters is shown in Table 1. The network is trained for 150k iterations with a batch size of 16, momentum 0.9, decay 0.005. The random scale is set within the range of [0.5,1.8] with a stride of 0.5. The learning rate (LR) is computed as,

$$LR=LR_{0}{\left(1-\frac{itr}{\max\_itr}\right)}^{0.9}$$ (2)

**Table 1:** Hyperparameters of learning process.

Hyperparameter	Value
Batch size	16
Epochs	16
Learning rate	0.0005
Momentum	0.9
Decay	0.005
Input image size	512 x 512
Kernel size	3 x 3
Dilation Rate	6
Number of classes	14
Activation Function	ReLU
Optimizer	Adam optimizer
Regularization	L2 Regularization
Normalization	Batch Normalization

Here *itr* is number of iterations, max_*itr* represents the maximum number of iterations which is set to 150. The initial learning rate (*LR*_0_) is set to 0.02. The combination of dropout, regularization, weight decay and early stopping and attention mechanism handles and reduces overfitting.

The Mean Intersection over Union (MIoU) is the mean result obtained by adding the ratios of the intersection and union of the anticipated outcome of each class and the true values, whereas the Overall Accuracy (Acc%) is a percentage corresponding to the number of pixels accurately identified to the total number of pixels.


$$MIoU=\frac{1}{N}\sum_{N}\frac{T_{-}P}{T_{-}P+F_{-}P+F_{-}N}$$ (3)

Where, N represents total number of class, *T*_*P* is the true positives where the number of pixels are correctly predicted. T_N is the total of true negatives, *F*_*P* is the false positives and F_N is the false negatives.


$$Acc\%=\frac{1}{N}\sum_{N}\frac{T_{-}P+T_{-}N}{T_{-}P+T_{-}N+F_{-}P+F_{-}N}$$ (4)

### 4.3 Results

**Fig 3:**
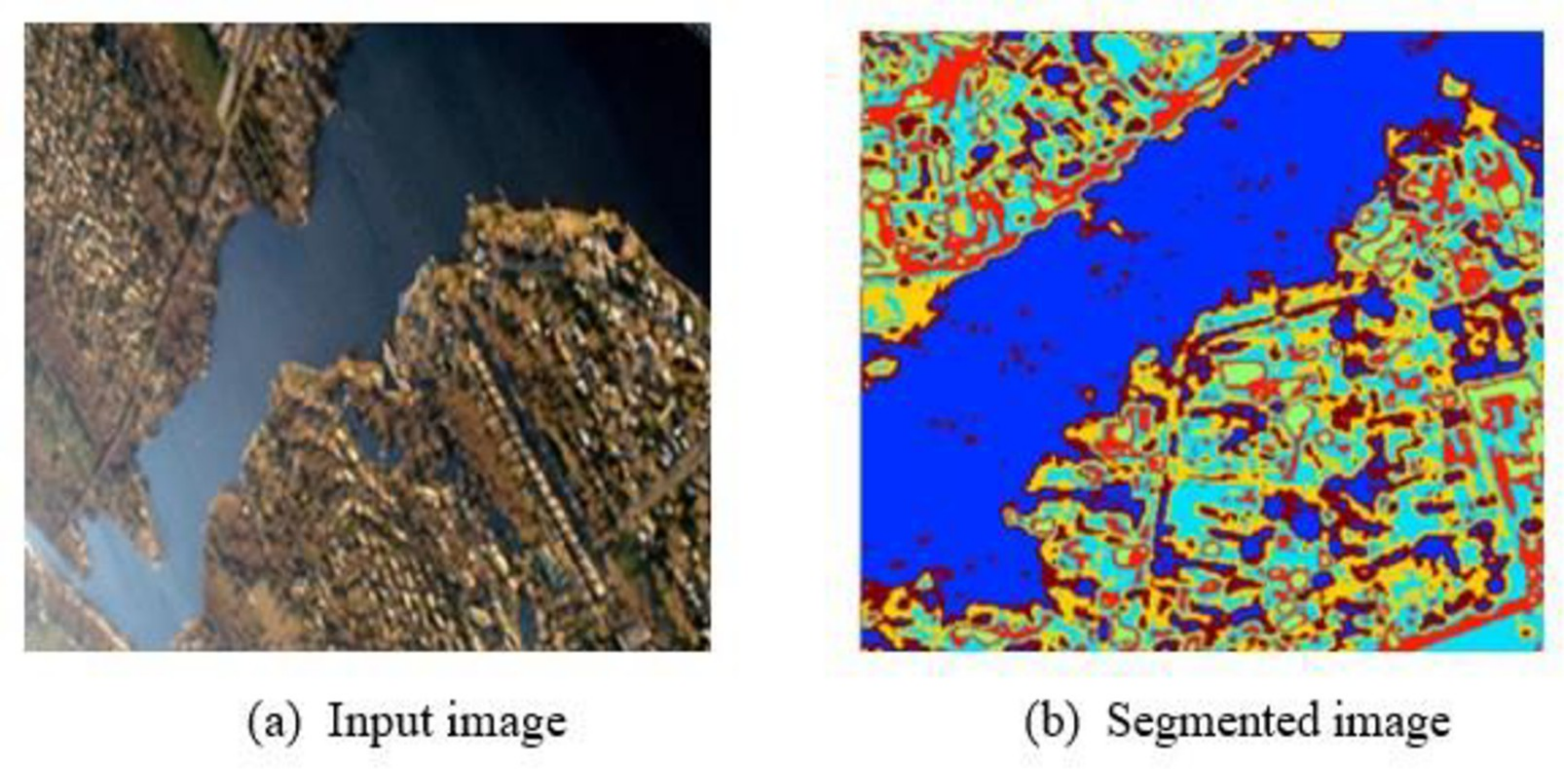
Segmentation Result (a) Input image (b) Segmented image.

The segmentation results obtained by the proposed FAttNet model is illustrated in Fig 3. The original input image is shown in **Fig 3(A)** and the segmented image is shown in **Fig 3(B)**.

**Fig 4:**
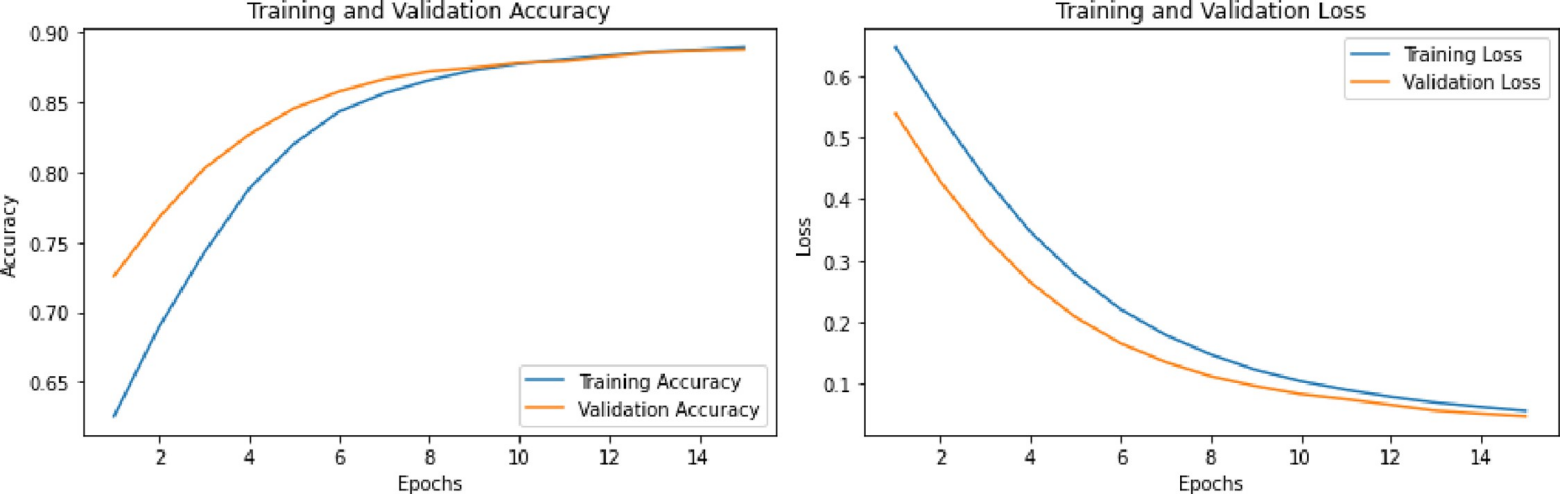
Epoch vs training and validation accuracy and loss.

As mentioned earlier, the quantitative assessment of segmentation outcomes for various networks involves estimating the mean Intersection over Union (MIoU) and segmentation accuracy. IoU is a metric that gauges the overlap between the predicted segmentation mask and the ground truth mask, with values ranging from 0 to 1—higher values signify better overlap. The MIoU, which calculates the average IoU across all classes, provides a class-specific measure of segmentation accuracy. The results of the mean Intersection over Union (MIoU) for various techniques on the GID dataset are presented in Table 2. Additionally, Table 3 and **Fig 4**. depict the overall accuracy achieved by the proposed method.

**Table 2:** IoU and mean IoU (MIoU) of Gaofen image dataset (GID).

Model	Farm area IoU	Forest area IoU	Water IoU	Fields IoU	Building IoU	Traffic IoU	MIoU
SegNet	58.58	77.87	76.34	74.94	52.03	62.76	67.09
Deeplab	65.03	78.79	80.89	75.12	62.05	66.85	71.46
Unet	62.86	79.78	81.04	74.26	59.21	62.63	69.96
ASPPNet	63.75	83.24	85.98	76.98	65.13	68.05	73.86
FAMNe2t	68.07	79.94	83.04	75.65	57.88	66.01	71.77
ACFNet	66.03	79.05	83.53	77.04	64.87	66.07	72.77
FAttNet	73.87	83.68	88.75	80.21	66.58	70.68	77.30

**Table 3:** Overall accuracy.

Model	Accuracy (%)
SegNet [19]	77.25
Deeplab [36]	79.48
Unet [21]	81.56
ASPP Net [37]	82.34
FAMNet [3]	84.87
MSCSANet [38]	83.76
ACFNet [39]	82.31
FAttNet (proposed)	87.26

Table 2 highlights the performance of different models on various land-cover categories, with FAttNet achieving the highest overall MIoU. ASPPNet and ACFNet also perform well, with MIoUs of 73.86 and 72.77 respectively, showcasing the effectiveness of attention mechanisms and spatial pyramid pooling in enhancing segmentation accuracy. Deeplab and FAMNet perform moderately, with MIoUs of 71.46 and 71.77, respectively, suggesting that while they are effective, they do not capture as much contextual information as FAttNet. Unet and SegNet show lower MIoU scores, 69.96 and 67.09 respectively, reflecting their limitations in dealing with high-resolution images and complex land-cover classes without the advanced attention mechanisms. FAttNet outperforms all other models with an MIoU of 77.30, indicating its robustness in segmenting high-resolution remote sensing images. It achieves the highest segmentation accuracy in all categories, particularly excelling in water (88.75), farm areas (73.87), and forest areas (83.68).

Across all models, the segmentation accuracy for water bodies is generally high, indicating that water bodies are relatively easier to segment due to their distinct spectral characteristics. Segmentation of buildings and traffic areas remains challenging across all models, as indicated by lower MIoU scores, reflecting the complexity and variability of these categories in high-resolution images. The superior performance of FAttNet highlights the significance of the class-aware feature attention mechanism in improving segmentation outcomes. By explicitly modeling the relationships between classes and feature channels, FAttNet can better capture contextually relevant information, leading to more accurate segmentation.

FAttNet achieves the highest overall accuracy of 87.26%, significantly outperforming all other models. This demonstrates the effectiveness of the class-aware feature attention mechanism in improving the segmentation accuracy of high-resolution remote sensing images. ASPP Net and ACFNet both show commendable results with accuracies of 82.34% and 82.31%, respectively, highlighting their capability to effectively segment remote sensing images, although slightly less effective than FAMNet. Unet performs well with an accuracy of 81.56%, showcasing its reliability as a segmentation model but revealing its limitations in achieving the highest performance. Deeplab and SegNet show lower accuracies of 79.48% and 77.25%, respectively, suggesting that while they are competent models, they do not match the advanced capabilities of the newer architectures with attention mechanisms. Fig 4 illustrates the training and validation accuracy and loss curves of the proposed model.

The significant improvement in accuracy with FAttNet and other top-performing models (FAMNet, ASPP Net, ACFNet) underscores the impact of integrating sophisticated mechanisms like attention mechanisms and spatial pyramid pooling. These enhancements allow the models to capture more detailed and contextually relevant features, leading to better segmentation accuracy. The overall accuracy results emphasize the advancements made by integrating attention mechanisms into segmentation models. FAttNet’s superior performance demonstrates that the class-aware feature attention mechanism is highly effective in enhancing segmentation accuracy.

## 5. Conclusion

The integration of a class aware feature-based attention approach into the semantic segmentation model for hyperspectral remote sensing images marks a significant advancement in capturing intricate details and enhancing the model’s ability to discern specific class features. The proposed study offers an upgraded Deeplabv3+ network that employs the FAttNet to semantically segment hyperspectral images. The FAttNet module can assist the network in learning diverse semantic information, while the spatial pyramid attention module can assist the network in learning multi-scale information. The proposed method enhances the segmentation accuracy and provides a more nuanced understanding of the observed scenes. The attention mechanism successfully directs the model to focus on relevant details, enabling precise identification and delineation of objects and land cover types. This advancement holds significant promise for applications such as land cover mapping, urban planning, and environmental monitoring. In the context of high-resolution remote sensing images, FAttNet utilizes the encoder-decoder structure to augment the segmentation results, leading to improved accuracy in segmentation. Investigations on the dataset validate the proposed FAttNet, which is then compared against many standard approaches.

Basic encoder-decoder architectures failed to balance deep semantic information with shallow spatial details, resulting in suboptimal segmentation outcomes. The optimization of the encoder-decoder structure in FAttNet ensured better integration of deep and shallow features, improving the accuracy and quality of segmentation, especially in the margin regions. This research introduces a novel class-aware feature attention mechanism that explicitly models these relationships. This mechanism allows for the extraction of contextually relevant data based on class information, significantly enhancing segmentation accuracy and robustness in hyperspectral images. The proposed method demonstrates superior performance by achieving a mean Intersection over Union (mIoU) of 77.03% and an accuracy of 87.26% on the GaoFen image dataset, outperforming existing mainstream segmentation networks.

The performance of the model is sensitive to hyperparameters, such as attention weights and feature transformation parameters. Fine-tuning is crucial to achieving optimal results, and a more automated approach to hyperparameter tuning should be explored. The additional computational demands introduced by the class feature-based attention mechanism may result in increased processing time and resource requirements. Balancing computational efficiency while maintaining high performance is a key challenge. As a result, future work could investigate automated hyperparameter tuning methods to streamline the process and ensure optimal performance across different datasets and scenarios. Optimization strategies shall be explored to mitigate computational overhead, making the method more suitable for real-time applications and resource-constrained environments consisting of varying terrains and landscape characteristics.
